# Using the magnetoencephalogram to noninvasively measure magnetite in the living human brain

**DOI:** 10.1002/hbm.24477

**Published:** 2018-11-20

**Authors:** Sheraz Khan, David Cohen

**Affiliations:** ^1^ Radiology Massachusetts General Hospital, Harvard Medical School Boston Massachusetts; ^2^ Athinoula A. Martinos Center for Biomedical Imaging Boston Massachusetts; ^3^ Francis Bitter Magnet Lab, Massachusetts Institute of Technology Cambridge Massachusetts

**Keywords:** brain aging, MEG, MEG methods development

## Abstract

During the past several decades there has been much interest in the existence of magnetite particles in the human brain and their accumulation with age. These particles also appear to play an important role in neurodegenerative diseases of the brain. However, up to now the amount and distribution of these particles has been measured only in post‐mortem brain tissue. Although in‐vivo MRI measurements do show iron compounds generally, MRI cannot separate them according to their magnetic phases, which are associated with their chemical interactions. In contrast, we here offer a new noninvasive, in‐vivo method which is selectively sensitive only to particles which can be strongly magnetized. We magnetize these particles with a strong magnetic field through the head, and then measure the resulting magnetic fields, using the dcMagnetoencephalogram (dcMEG). From these data, the mass and locations of the particles can be estimated, using a distributed inverse solution. To test the method, we measured 11 healthy male subjects (ages 19–89 year). Accumulation of magnetite, in the hippocampal formation or nearby structures, was observed in the older men. These in‐vivo findings agree with reports of post‐mortem measurements of their locations, and of their accumulation with age. Thus, our findings allow in‐vivo measurement of magnetite in the human brain, and possibly open the door for new studies of neurodegenerative diseases of the brain.

## 1 INTRODUCTION

Magnetite (Fe_3_O_4_) particles in the human brain were first reported in 1992 (Kirschvink, Kobayashi‐Kirschvink, & Woodford, 1992a), where crystallography, electron, and x‐ray diffraction were used to identify these particles in the post‐mortem brain. These were found to have a range of sizes, from superparamagnetic to single domain (Bozorth, 1993; Jiles, 2015; Nagata, 1961). This first work was succeeded by further studies of magnetite in the normal brain (Kirschvink, Kobayashi‐Kirschvink, Diaz‐Ricci, & Kirschvink, 1992b; Maher et al., 2016) including the distribution (Gilder et al., 2018) and effect of age (Dobson, 2002), and many studies of magnetite in brains with neurodegenerative disease (Castellani et al., 2007; Collingwood & Dobson, 2006; Collingwood & Telling, 2016; Dobson, 2001, 2004; Grünblatt, Bartl, & Riederer, 2011; Hautot, Pankhurst, Khan, & Dobson, 2003; Pankhurst, Hautot, Khan, & Dobson, 2008; Plascencia‐Villa et al., 2016; Quintana et al., 2006; Scaiano, Monahan, & Renaud, 1997; Smith, Harris, Sayre, & Perry, 1997; Smith et al., 2010; Teller, Tahirbegi, Mir, Samitier, & Soriano, 2015).

There has been much interest in magnetite because of its strong interaction with elements in the brain, due to its combination of redox activity (Everett et al., 2014), strong magnetic behavior, particle surface charge (Grünblatt et al., 2011), and Fenton‐like chemistry (Collingwood & Telling, 2016). Some authors believe that magnetite serves a physiological purpose in the normal brain (Kirschvink, Kobayashi‐Kirschvink, A., Diaz‐Ricci, et al., 1992b). However, it has now been established that magnetite is strongly associated with degenerative diseases of the brain; where accumulation of these particles in the brain appears to be increased (Dobson, 2004). This is especially true in Alzheimer's disease, where magnetite nanoparticles were found associated with tangles and plaques (Collingwood & Dobson, 2006; Everett et al., 2018; Plascencia‐Villa et al., 2016).

However, these studies were always carried out in the post‐mortem brain. Although many noninvasive MRI studies of iron oxides were indeed performed in the living brain (Bartzokis et al., 2000; Collingwood et al., 2005, 2008; Daugherty & Raz, 2015; Gossuin et al., 2005; Langkammer et al., 2010; Yan, Sun, Yan, Wang, & Lou, 2012) showing iron oxides generally, magnetite could not be separated out selectively. Therefore, estimation of magnetite mass and distribution, which can be important in the understanding of neurodegenerative diseases of the brain, up to now cannot be made in the living brain.

In contrast, we here offer a noninvasive method for indeed measuring magnetite particles in the living brain. In brief, we magnetize these particles by a strong magnetic field through the head, and then, in a magnetically‐shielded room, measure the resulting dc magnetic fields over the head produced by the magnetized particles. To do this, we use the dcMagnetoencephalogram (dcMEG), which is based on detectors called superconducting quantum interference devices (SQUIDs). From these magnetic field data, the mass and distribution of the particles within the head can be estimated. Thus, our method depends on using “isothermal remanent magnetization” (Bozorth, 1993; Jiles, 2015; Nagata, 1961) of the particles in the living head. Our method of measuring magnetite particles is a new and alternate use of the dcMEG, which normally measures the magnetic field due to dc current sources, not magnetic particles.

In review, the fluctuating (ac) magnetic fields produced by the human body are well known. For example, the weak ac magnetic field produced by the human heart (the magnetocardiogram) (Baule & McFee, 1963) and that produced by the human brain (measured by the MEG) (Cohen, 1972) have been well studied. Further, the steady (dc) magnetic field of the human body has also been studied (Cohen, 2004). One old dc report was a mapping of the dc field over the entire normal body (Cohen, Palti, Cuffin, & Schmid, 1980). Other reports were of dc from the ischemic heart (Savard, Cohen, Lepeschkin, Cuffin, & Madias, 1983), and dc from various states of the brain (Bowyer et al., 2012; Leistner et al., 2007; Sander et al., 2007).

In the old dc mapping over the body, only one source of the dc field over the head was seen, which was a new phenomenon: when the scalp was lightly pressed at a region containing healthy hair follicles, an external dc field appeared over that region. We note that this field could be dc interference in this present study. However, in that early work, no dc fields from ferromagnetic particles were seen; the sources were always dc currents. Otherwise, magnetite particles in the lungs have been extensively studied separately (Cohen & Nemoto, 1984; Cohen, Nemoto, Kaufman, & Arai, 1984).

More recently, we have returned to the old work (Cohen et al., 1980) and further studied the dc magnetic field over the head, but this time using the advanced dcMEG. The detector in the old work consisted of only one pair of pickup coils called a double planar gradiometer (two SQUIDs). It was located in the tail of a large dewar and measured only at one location at a time; therefore, a dc mapping over the head was most cumbersome. But with our present state‐of‐the‐art MEG system we now have 102 planar gradiometer pairs (204 SQUIDs), in a helmet spaced over the entire head, and one scan measures the entire head, resulting in an “arrowmap” over the head.

With this new system, we again saw, and studied the dc from the hair follicles (Khan & Cohen, 2016). But now we also saw some new phenomena in the head, including some new, comparatively small dc currents called “wings.” We also saw dc magnetic fields produced by ferromagnetic material in the head, for the first time. This aroused our interest because of its disease‐diagnostic possibilities, leading to this present work.

Our method only shows material in the brain which can be strongly magnetized, that is, which is ferri and ferromagnetic, which allows only two compounds: magnetite and maghemite (Fe_3_0_4_ and γFe_2_O_3_), the strongly magnetized compounds of iron. The compounds of the other magnetic elements, nickel and cobalt, are not significantly present. Maghemite was originally seen along with magnetite (Kirschvink, Kobayashi‐Kirschvink, & Woodford, 1992a), but appears to play only a small role in comparison to magnetite (Collingwood & Telling, 2016). Maghemite has about the same magnetization curve as magnetite, and in our system, we cannot observe the difference between the two compounds. We, therefore, focus on magnetite, with the understanding that a lesser amount of maghemite is probably also involved but need not be further mentioned.

To test the method, we measured 11 healthy male subjects, of ages 19–89 years, who are mostly bald or thin‐haired (to minimize the follicle artifact). We compare accumulation of magnetite with age to the reported in‐vitro findings (Dobson, 2002), to verify our method.

## 2 MATERIALS AND METHODS

**Figure 1:**
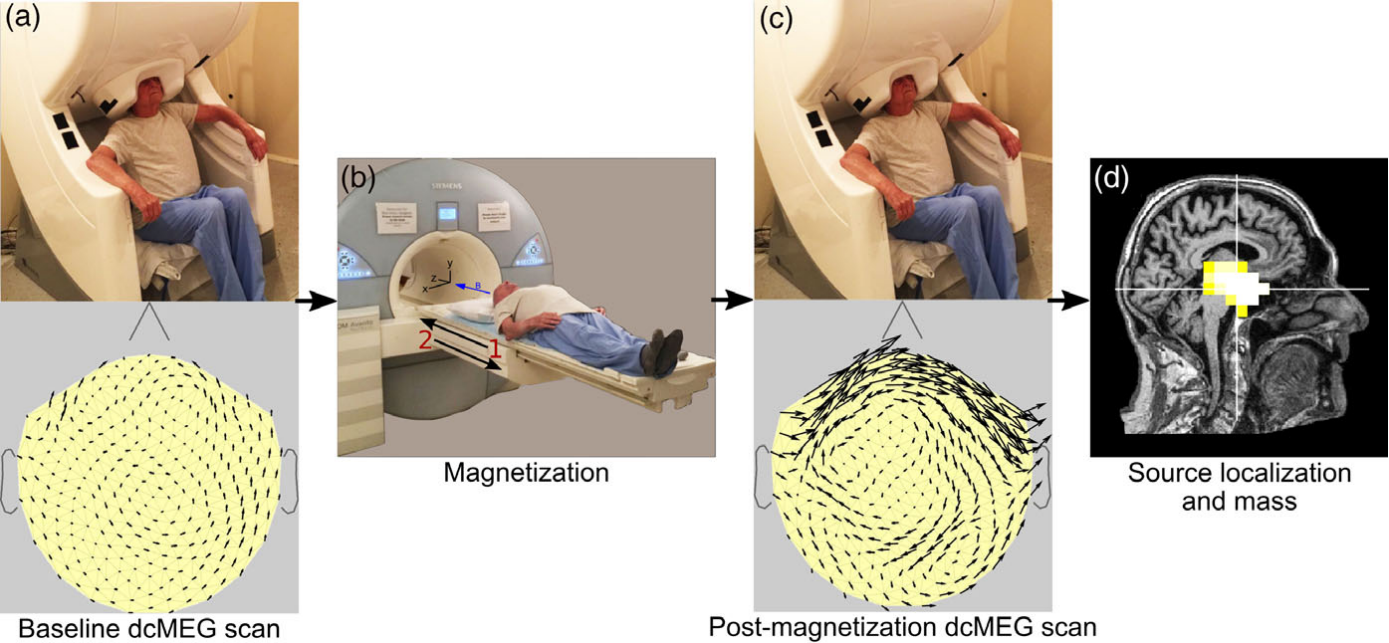
Schematic illustration of four steps of the method. (a) Baseline dcMEG scan. (b) Magnetization. (c) Post magnetization dcMEG scan. (d) Source localization and mass [Color figure can be viewed at http://wileyonlinelibrary.com]

This method consists of four main steps, as illustrated in Figure 1. We will explain each step in detail.

### 2.1 Step 1: Baseline dcMEG scan

#### 2.1.1 Preliminary information: The dcMEG

**Figure 2:**
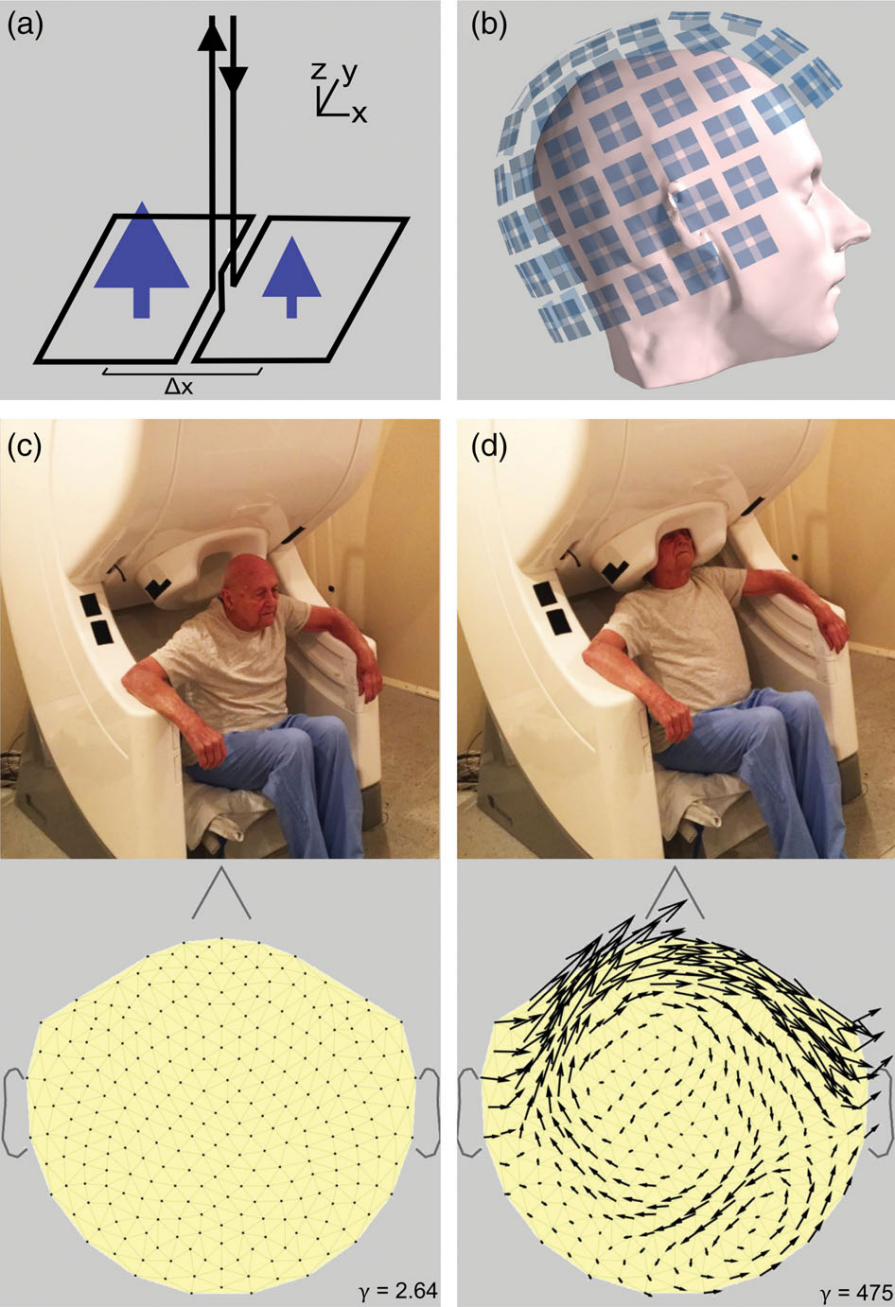
a and b: Geometry of the gradiometers in our dcMEG. (a): Superconducting wire loops of a single planar gradiometer. Blue arrows are average normal magnetic field *B*_*z*_ through each loop. The gradiometer output is proportional to Δ*B*_*z*_/Δx. The other half of a gradiometer pair is obtained by rotating the arrangement by 90^0^, not shown here, yielding the second output Δ*B*_*z*_/Δy. The coordinate system is fixed to the coil, in this drawing. (b): The arrangement, inside the liquid‐helium dewar (helmet) of 102 gradiometer pairs around the head. c and d: The dcMEG measurement. (c) Upper panel: A subject at the dcMEG, with the head outside the helmet. Lower panel: Arrowmap, looking down on the head, has just been zeroed, for his head in this outside position. (d) Upper panel: The head has just been put inside the helmet. Lower panel: Resulting arrowmap, showing dc (atomic currents in the magnets) due to this new head position [Color figure can be viewed at http://wileyonlinelibrary.com]

This preliminary information will be used in steps 1 and 3. Our MEG was manufactured by the Elekta Co. and is the model called VectorView, which needs to be configured for dc. The later model from Elekta is just as suitable. These models contain 306 SQUID detectors, arranged in groups of three, at 102 locations over the head, where each SQUID is fed by a pickup coil sensing the ambient field. There are therefore three pickup coils at each of the 102 locations. One coil is a magnetometer (simple loop) and the other two coils make up a planar gradiometer pair. We use only the gradiometers, that is, 204 SQUIDs, not the 102 magnetometers. This is a large advance over the original old system. The gradiometer set‐up is shown in Figure 2. To convert to dcMEG, we set the lower bandwidth limit down to 0 Hz and arranged the gradiometer outputs to be displayed as a map of arrows over the head. Measurement results with this system appear as “arrowmaps,” for example, as in Figure 2d (lower panel), where each arrow represents the dc current underneath. These maps allow visual on‐line determination of magnetite sources.

For any dcMEG measurement, the subject first rests his head outside the helmet, and the arrows are zeroed, as in Figure 2c. Then, as in Figure 2d, the subject puts his head into the helmet, lightly touches the top of his head against the inner helmet, and the new arrows (dc currents) are seen and recorded. He then removes his head again, and the arrows return to near‐zero again.

In configuring the MEG for dc, we chose the gradiometer‐based system over a magnetometer‐based system because of the fluctuating dc field background. The gradiometer‐based system is normally immune to this fluctuation, especially in our heavily shielded room (Cohen, Schläpfer, Ahlfors, Hämäläinen, & Halgren, 2002).

#### 2.1.2 Preliminary information: Setting up the arrows

This preliminary section is necessary in steps 1, 3, and 4. We here set up the arrowmap. We note that the original gradiometers take the gradient in a spherical coordinate system, that is, along latitude and longitude. First, they are mapped by us to the virtual gradiometers, taking the gradient in Cartesian coordinate system. The Hosaka–Cohen transformation (Cohen & Hosaka, 1976) is then applied on these gradiometers:

$$\vec{v_{n}}=\frac{\partial B_{z}}{\partial y}{\hat{x}}_{n}-\frac{\partial B_{z}}{\partial x}\;{\hat{y}}_{n}$$

where, $\vec{v_{n}}$ are the transformed gradients (Hosaka–Cohen transformation) at each location, *B*_*z*_ is the *z* component of the magnetic field, $\frac{\partial B_{z}}{\partial y}$ and $\frac{\partial B_{z}}{\partial x}$ are the output of the virtual planar gradiometer, and ${\hat{x}}_{n}\;$and ${\hat{y}}_{n}$ are the unit orientation vectors. To quantify the total magnetic (gradient) strength in detector space, we define γ as the sum of the lengths of the arrows in the arrowmap:

$$\gamma =\sum_{n=1}^{N}\sqrt{{x_{n}}^{2}+{y_{n}}^{2}}$$

where, *x*_*n*_ and *y*_*n*_ are the x and y component of the arrow $\vec{v_{n}}$, the square root of each amplitude squared gives the amplitude of each arrow, and *N* is the total number of arrows in the map. Further, to subtract one map from another, we define Δγ, as the difference between map #1 (Figure 1a) and #2 (Figure 1c), as an example:

$$\Delta \gamma =\sum_{n=1}^{N}\sqrt{{\left(x_{n_{1}}-x_{n_{2}}\right)}^{2}+{\left(y_{n_{1}}-y_{n_{2}}\right)}^{2}}$$

The subtraction is needed to possibly subtract out the baseline map with unwanted signals. γ is simply proportional to the gradient fT/cm. γ is calculated for every map in this work, for example 475 in Figure 2d lower. We note that γ for Figure 2c lower is not zero, but a small number 2.64, showing the noise, a second or two after the reset producing true zero.

γ is used in the following way. We find the two possible quantities which produce the arrowmap: (1) current dipoles, such as dc in the scalp due to hair follicles; (2) magnetic dipoles, such as magnetite, or (unwanted) magnetic dental work. Therefore, there are two calibrations that should be done. Use a known (point) current dipole as the source, at a known distance *z* down into the head (from the air‐fiberglass interface) at a known orientation; or a known (point) magnetic dipole at a known *z* and orientation and note the γ for each. For best accuracy, *z* should be an approximate match to where we expect our actual source to be, in each case. For the work presented here, we almost completely deal with second case, magnetic dipoles, and mostly ignore the first case, current dipoles.

It can be shown that each arrow has the units kμA/cm^2^, where k is a constant of proportionality and μA is microampere. Each arrow is therefore proportional to the local current density. If the source is an internal dc bio‐current, then the arrow indicates that underlying current. If, on the other hand, the source is a magnetic particle, then an arrow is again a current, but is the internal atomic current producing ferromagnetism.

#### 2.1.3 Actual step 1

In preparation for the first step, the subject changes into nonmagnetic clothes, and his scalp and/or hair is washed, to remove any artifactual ferromagnetic particles, common in the dust of an urban environment. This first step, seen in Figure 1a, is a baseline dcMEG scan, before any deliberate magnetization, to see any previously‐magnetized material which might exist in the head, perhaps due to a previous MRI; or to see any ferrous dental work, which could produce enormous signals, even with small magnetization. The baseline scan appears in the form of an arrowmap over the head, as for all dcMEG scans in this work. In most subjects of this study, the arrow signals in the baseline scan have been manageable or negligible. However, in a few cases, it was necessary to subtract baseline arrowmaps from remanent arrowmaps, to see the “true remanence.” This would be due, for example, to natural dc sources in the head, such as healthy hair follicles (see Figure 5 ahead).

### 2.2 Step 2: Magnetization

The second step is magnetizing the magnetite particles. That is, we apply maximum H, in the B–H curve. For convenience, to perform the magnetization, we use the superconducting magnet of a nearby 1.5 T MRI scanner. The subject is slid into the scanner so that the omni‐present strong uniform magnetic field is applied to the head, which magnetizes the particles well into saturation. Then the field is removed, by sliding the subject out of the field. That is, we reduce H to almost zero, in the B–H curve. Concerning the length of time of magnetization, we have always used 20 s or more, and have never seen signs of under‐magnetization. When an MRI is performed on some subjects to get an MRI image (necessary for step 4), the extra exposure time to the magnetic field of about 6 min, produces no measurable effect. It therefore appears that saturation takes place in less than 20 s, perhaps in much less time.

Figure 1b shows preparation for magnetization. A subject from our study is prepared, far out in the fringing field, to be slid deep into the MRI to magnetize any magnetite in his head, in the +*z*‐direction. It is important that this direction of magnetization, in the head, be maintained when the subject is being slid in and out, in line with the blue axial magnetic vector B; otherwise there would be some magnetization in an off‐*z* direction. With some subjects, who have no prior MRI of the head, this is also an opportunity to record a T1 MRI of the head, used for the final display of the particles locations (Figure 1d). The arrows 1 and 2 show how the patient is rolled into and out of the magnet center. In principle the toe‐to‐head axis of the body should always be aligned with the axis of the magnetizing coil, but this is not quite possible in the fringing field of the large magnets, because the subject approaches standing up, so we approximate as best we can. If the subject is placed out on his bed quite far out, there does not seem to be a problem. However, if the subject is placed as he would be in an ordinary MRI medical head scan, close in to the magnet, we found that the inverse solution is degraded, because there was too much magnetization in the y‐direction.

Because the subject is moved through the *z*‐gradient of the fringing field, there is a translational *z*‐force on each particle, as noted by (Dobson, Bowtell, Garcia‐Prieto, & Pankhurst, 2009). No effect of this force has been seen by us, at least with the 1.5 T magnet, and we can ignore this phenomenon.

### 2.3 Step 3: Post magnetization dcMEG scan

The preliminary information from step 1 is used here. After magnetization, the subject is quickly walked back to the shielded room, in order to minimize any relaxation of the magnetite. The subject is then measured with the. dcMEG, at a time 3 ± 0.5 min after magnetization, producing the main data arrowmap of remanent magnetization. The exact way each particle has changed, during the magnetization, depends on the particle size, shape, and viscous environment in a complex way. Our method is effective over a wide range of these parameters. However, in principle, we can lose some remanence, or experience relaxation, in several ways. If the particles are of small size, say less than 10 nm, after removal of the magnetizing field, they can be rapidly rotated out of alignment by thermal collision, by superparamagnetism (Jiles, 2015). Or, after magnetization, there can be relaxation if the viscosity of the medium behaves to elastically reverse the particle motions.

To allow co‐registration of the MEG and MRI image of the subject, for localization, the positions of three fiduciary points (nasion and auricular points) that define a head‐based coordinate system, a set of points from the head surface, and the locations of the four Head Position Indicator (HPI) coils were determined. We used a Fastrak digitizer (Polhemus Inc., Colchester, VT) integrated with the VectorView system. The position and orientation of the head with respect to the MEG sensor array were also recorded with the help of these HPI coils.

Next, 15–20 min after the 3‐min recording, a new arrowmap was recorded, to see any particle relaxation. With some older subjects, recordings were made 1 day, 2 days, etc., and even weeks later, to see further relaxation, if any.

### 2.4 Step 4: Inverse solution: Solving for location and total mass of magnetite

#### 2.4.1 Preliminary information: Calibration sample

To find the mass, we used a calibration curve. That is, we placed a known mass of fully (saturated) magnetized magnetite at a number of *z*‐locations and rotational angles in the helmet, and recorded the resulting arrowmaps, including the γ^′^s. We used a calibration magnetite sample prepared at the Prof, Joe Brain dust lab at the Harvard School of Public Health. From their Pfizer MO‐7029 sample of magnetic iron oxide (Fe_3_O_4_, bottle dated September 9, 1980), described as cubical magnetite, (also as cynical with diameter 0.5 μm), 8.7 mg iron oxide was weighed and diluted to 1 mg/mL in ddH_2_O (distilled deionized water). This was briefly sonicated. Meanwhile, 0.16 g agarose was dissolved in 10 mL ddH_2_O at about 80°C to make a roughly 1.6% solution of agarose.

The iron oxide was vortexed thoroughly and 10 μL immediately withdrawn by micropipette and put in a 15 mL tube and topped with 5 mL hot agarose solution from a 5 mL micropipette. This was pipetted up and down to mix and split between two pieces of clear packing tape, about 4 cm wide, to make a roughly 3 cm circle on each. (Each of these circles contains half of the original 10 μL of 1 μg/μL iron oxide, so there are 5 μg iron oxide in each.) This was then allowed to dry overnight. The dried spots were flat but caused the tape to curl.

The samples were topped with pieces of 4 cm wide masking tape, and in an effort to flatten them properly they were then surrounded with two more thicknesses of masking tape. These were labeled in pencil (and the pencil label covered in Scotch tape).

**Figure 3:**
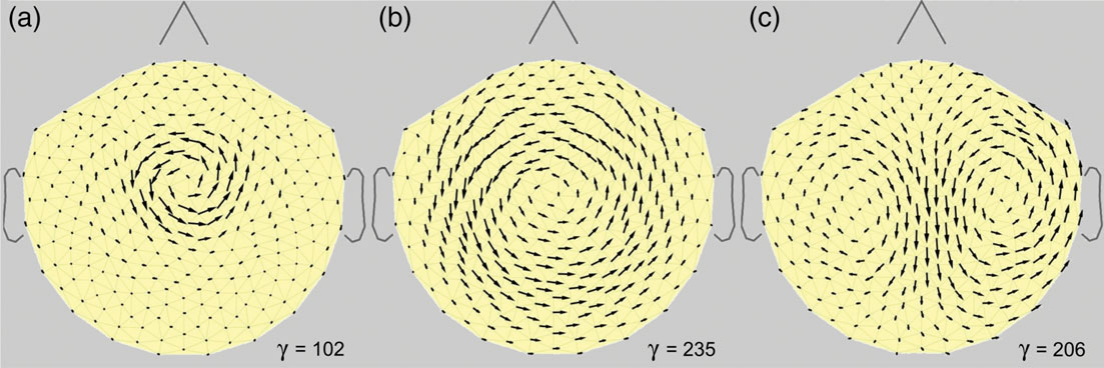
Calibration arrowmaps due to a 5‐μg sample of magnetite dust, placed at various *z*‐distances along a central radius, and oriented at two different angles. (a): *Z* = 0 cm, θ = 0°. (b): *Z* = 7.0 cm. θ = 0°. (c): *Z* = 7.0 cm, θ = 90^0^. *Z* = 0 is located at the air‐fiberglass interface, *z* = 7.0 is at location of most particle inverse locations. The gain in (a) has been reduced a factor of 10, compared with the gain in (b) and (c), because the arrows are so large, at this *z*; thus γ is actually 1,020. Θ is the angle between the dipole and the *z*‐axis. We ignore the second angle, because the first angle Θ makes almost no difference to γ . By turning through various angles, the pattern in c becomes, for example, close to the arrowmap recorded pattern in Figure 2d lower [Color figure can be viewed at http://wileyonlinelibrary.com]

Some of the calibration arrowmaps (of the many we recorded for visual study) are shown in Figure 3. From these maps, we learned how to estimate the magnetite distributions in the actual subjects, by looking at their arrowmaps on‐line, to make rapid recording decisions (e.g., should we wash his head again?) The subjects' patterns can also be calculated by forward modeling, as we have done, to lend confidence.

#### 2.4.2 Preliminary information: The calibration curve

**Figure 4:**
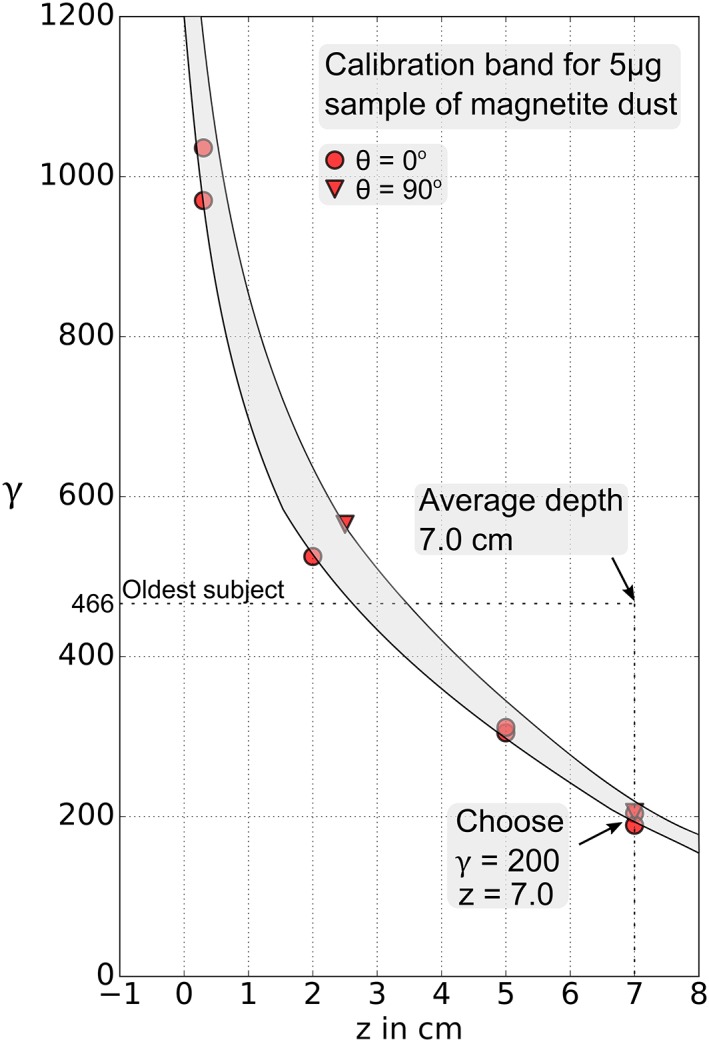
Calibration curve (band), used to find the total magnetite mass, for the 89 y/o subject. To begin, the inverse solution has shown an average depth of the particle “clump” for this subject of 7.0 cm. Thus, a broken horizontal line is made at γ = 466, and it is run rightward till *z* = 7.0. Then it is run down vertically, and γ =200 chosen from the band. That indicates a mass of 466/200 x 5.0 = 11.7 μg for this subject. That is, 466/200 = 2.3 calibration samples to produce the same γ [Color figure can be viewed at http://wileyonlinelibrary.com]

This preliminary is necessary in step 4. Our calibration curve is γ versus *z*, at a particular dipole angle, for 5 μg of magnetite dust. The calibration curves at different angles fortuitously are within 10% of each other, from which we made a master curve, and accept a 5% maximum error due to angle. The calibration curve we used is shown in Figure 4. From this curve we will find the mass which produced the arrowmap. We make a horizontal line at the appropriate γ value, and run it out horizontally to, say, *z* = 7 cm. We then ask: how many samples of 5 μg, to reach that point vertically? For example, in this case it is 2.2 samples, yielding a total clump mass of 11.7 μg, for the 89 y/o subject.

The curve (actually a band) is not meant to be accurate, but only to be approximate, as a first trial of this new method. We accept the errors, for the time being. The goal is to refine the calibration later. The band is made the following way: the red dots are 6 measurements of γ versus *z*, for the 5 μg magnetite sample, where *z* is the distance from the helmet–air interface, down toward the head center all at θ = 0°; that is, the 3‐cm disc of magnetite dust is perpendicular to the *z*‐axis. The red triangles are three measurements where we draw the best curve through the six dots, and another (higher) curve through the three triangles, guided by the lower curve. We then fill the space between with a gray band. There is about a 90% probability, that for any given *z*, the true value is within the band. The red dots have really been moved upward to account for some few % of relaxation which has been seen in the sample, over the several days of measurement, after magnetization. This may be due to the presence of some multi‐domain particles. The band denotes an error of about ±8% which, again, is acceptable for the time being, on top of other errors.

Note that this sample is spread over a 3‐cm diameter, hence a correction is needed. However, the gradiometer coils are about 20 mm in from the air interface. All in all, for the entire spacing picture, the error of this 3‐cm spread lies within the gray band, and we here make no special correction.

#### 2.4.3 Preliminary information: Forward model and distributed inverse solution

This preliminary is necessary in step‐4. The source locations of the magnetic dipoles were estimated from the gradiometer raw data in the following way. For the forward solution, the head model: T1‐weighted, high resolution magnetization prepared rapid gradient echo (MPRAGE) structural images were acquired on a 1.5 T Siemens whole‐body magnetic resonance (MRI) scanner (Siemens Medical Systems), using a 12‐ channel head coil.

The structural data of the head was preprocessed using FreeSurfer (Dale et al., 2000). To compute the forward solution, the skull was segmented using the watershed algorithm in FreeSurfer. Brain volume was then discretized in three‐dimensional volume source space bounded by the outer skull with a margin of 4 mm. Volume source space consisted of a 3D grid of 1,568 dipole, corresponding to a spacing of approximately 12 mm between adjacent source dipole locations. To compute the forward solution, a magnetic dipole was placed at each of the 1,568 3D grid locations.

The magnetic dipole distribution was estimated using the distributed solution approach employing minimum‐norm estimate (MNE) with free orientations. MNE estimates the sources as the solution to a linear imaging problem and estimates source density image jointly for all the dipoles that best fits the data and favors solutions that are of minimum energy (or L2 norm).

The regularized (regularization = 0.1) noise covariance matrix used to calculate the inverse operator was generated using empty shielded‐room data. The dynamical statistical parametric mapping (dSPM) (Dale et al., 2000) map was calculated by dividing MNE value with the projection of the estimated noise covariance matrix at each source point. For visualization in Figure 7, Supporting Information Figures S1–S3, dSPM maps were thresholded at 99% percentile of the source distribution.

We have used the forward equations to simulate the arrowmap patterns we can expect from simple angular arrangements of magnetite when they are a magnetic dipole. These are the patterns we get when the particles are clumped together in the brain, to a single location, magnetized in the +*z* (toe‐to‐head) direction, at several different locations. These will be guides in understanding actual patterns we get, in our study shown below, in Results. Thus, this produces the spatial distributions of the dipoles, responsible for the arrowmaps.

#### 2.4.4 The actual step 4

The fourth step consists of inverse modeling (also known as source localization) of the measured data, to estimate the mass and location of the particles. The distributed inverse solution, which estimate source density image jointly for all the dipoles, was performed as described earlier. Unlike parametric methods like single or multiple dipole fit which assumes sources can be represented by a few equivalent dipoles, the distributed solution estimates source density image jointly for all the dipoles.

The source space consisted of 1,568 dipoles uniformly distributed inside the skull. The source distribution was estimated using the minimum‐norm estimate (MNE) with loose orientation. The regularized (regularization = 0.1) noise covariance matrix used to calculate the inverse operator was from empty room MEG recording. The dynamical Statistical Parametric Mapping (dSPM) (Dale et al., 2000) map was calculated by dividing MNE value with the projection of the estimated noise covariance matrix onto each source point. The estimated source density from dSPM is then threshold to at 99% percentile of the source distribution for visualization. All forward and inverse calculations were done using MNE‐Python.

### 2.5 Artifacts

#### 2.5.1 Natural artifacts

**Figure 5:**
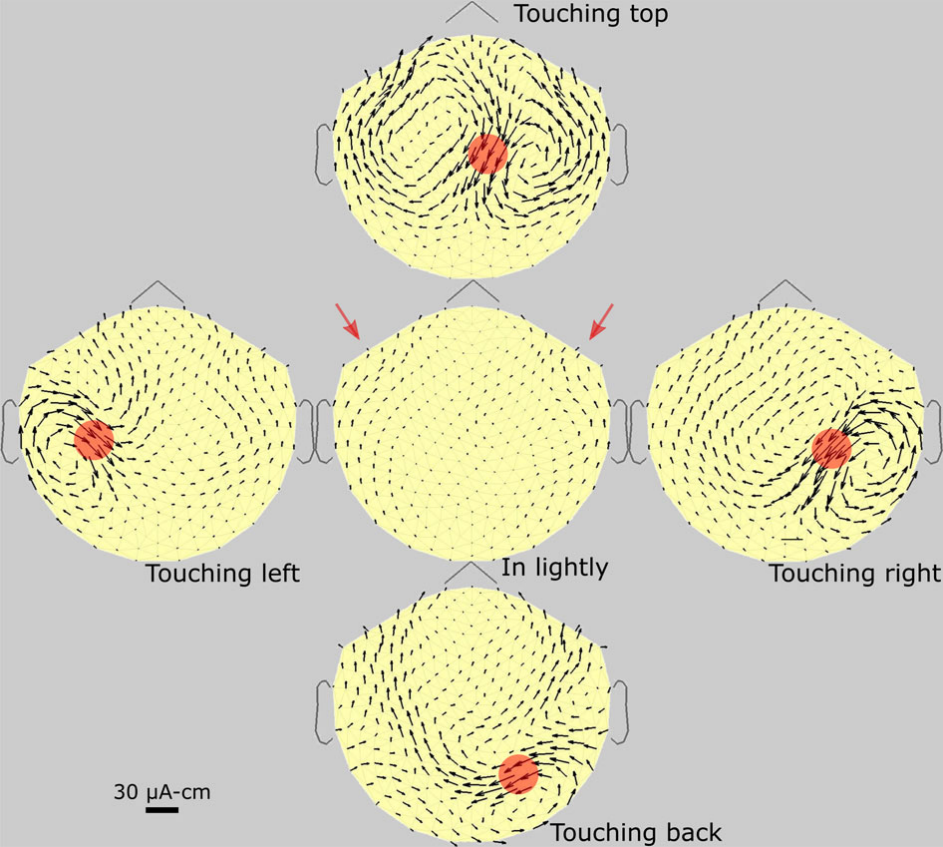
Set of arrowmaps which is our standard example of the dc field due to hair follicles. The subject is a 37 y/o full‐headed male subject. The red circles show the approximate areas of pressure. The black arrows under the circles are the dc sources or “batteries,” generating the resulting arrows in the resistive volume current of the scalp. The shapes of the arrow loops (currents) are determined by the variation of the shape and resistivity of the low‐resistance scalp, as well as by the angle of generating‐follicle tilt. The black bar refers to the battery strengths only, under the red circles. The largest “resistive” arrows, not under the circles, have a length of about 2 pT/cm, equivalent to about 1 μgm of magnetized magnetite at a depth of *z* = 7 cm. The “wings” (red arrows) are equivalent to about 0.2 μgm, at 7cm, essentially negligible [Color figure can be viewed at http://wileyonlinelibrary.com]

Our most common natural artifact is the dc due to pressing on healthy hair follicles, equally for men and women. We here briefly summarize this signal. The signal strength depends on the follicle density along the scalp, and their health. Different parts of the head can be pressed against the inner helmet wall, giving different patterns, as shown in Figure 5. The exact microscopic source mechanism is unknown. To minimize these interfering signals, we chose all men with as little hair as possible. Thus, one subject was almost completely bald, four subjects were about 60% bald, four subjects were about 20% bald, and two were full‐headed.

The next natural artifact is the “wings” in Figure 5, present in about 80% of subjects, but is essentially small and can be neglected in this first high‐error study. There are yet smaller artifactual dc signals from the head, but they are certainty below our signal thresholds.

#### 2.5.2 Human‐made artifacts

Aside from ferromagnetic dental work, the most common artifacts of this type are ferromagnetic dust particles in an urban setting, due to furnace smoke, and train wheels and rails. Because this dust is especially bothersome in the Boston area, all subjects have their hair washed, just before step one. But we have found that about 25% of step three measurements, just after magnetization, show magnetized dust contamination on their heads, easily recognized by arrowmap patterns. Therefore, we perform another quick head‐wash, followed by step three, again. For our next study, we plan on using a disposable paper hat during magnetization.

**Figure 6:**
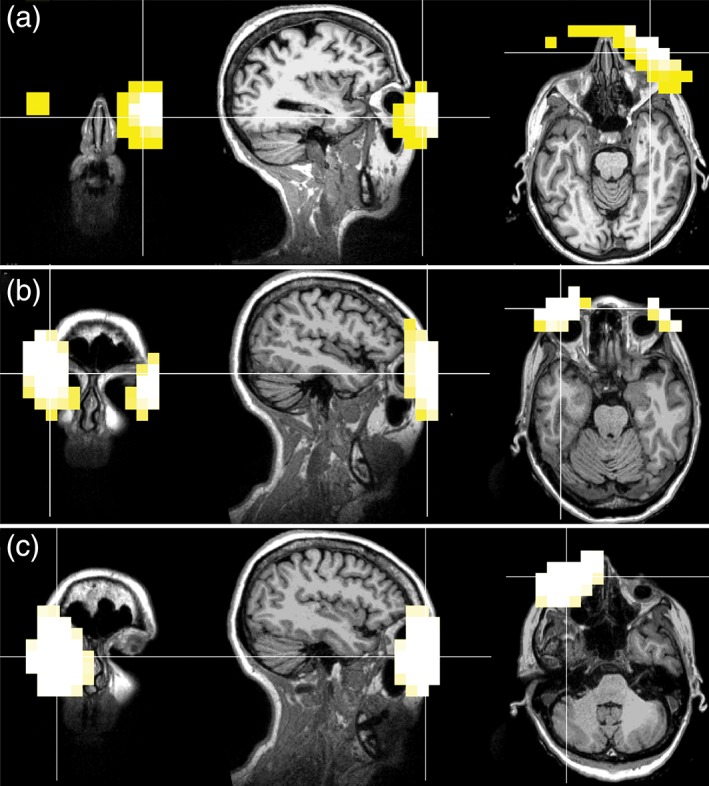
(a) Inverse solution of woman subject #1, showing ferromagnetic material located in her right upper front tooth, known to have an implant, her single implant. All other dental work consisted of routine fillings. (b) Inverse solution of woman subject #2, first measurement, showing ferromagnetic material (mascara) at both eyes. (c) Inverse solution of woman subject #2, second measurement, at higher sensitivity, 1 week later, after a number of attempts to clean off the mascara during the week. The right eye is seen to be clean of ferromagnetic contamination, but the left eye is still somewhat contaminated [Color figure can be viewed at http://wileyonlinelibrary.com]

Two other artifacts of this type were seen and dealt with, and unexpectedly gave us new localization information. Three separate measurements took place of two subjects who had ferromagnetic contamination in or on their head. These were two women subjects who were part of a program which began after we finished the sample of men recorded here. Woman # 1 had a recent tooth implant (upper right front); these are usually nonmagnetic, but in this case apparently a slightly ferromagnetic alloy was used. In other words, she presented with a contaminated tooth, perhaps with a total mass of 30μgm magnetite equivalent. Her inverse solution is seen in Figure 6a. Although we estimate the uncertainty to be ±1.5 cm, the method localizes more accurately, in this case.

Woman subject #2 showed ferromagnetic contamination in the form of mascara on the eyes, known to get its black color from magnetite. Figure 6b shows the inverse solution when both eyes were initially contaminated, then Figure 6c shows the solution after an attempt of a week of cleaning, still leaving one eye with a little contamination. Again, although we estimate the uncertainty to be ±1.5 cm, the localization is better, in these cases. The point here is that these contaminations are an unexpected test of the accuracy of this method. The accuracy is surprisingly good, for the beginning of a new technology.

### 2.6 Subjects

The participants were 11 adult males, in the age range of 19–80 y/o. The particular age distribution can be seen below, in Figure 8. Older subjects were mostly bald or thin haired. Informed consent approved by the institutional review board (IRB, Mass. General Hospital, Boston, MA) was obtained from all participants. All subjects were healthy, by which we mean that they presented with no obvious health problems, especially neurological problems. Also, they questioned at length and presented with no obvious ferromagnetic material in their mouths or body. Only the imaging of MEG and MRI were acquired from these patients, as were approved by the IRB. It was not necessary to perform CT scans to look for metallic (ferromagnetic) artifacts in the head, because the dcMEG can be much more sensitive, in this regard, and would easily respond to ferromagnetic artifacts not seen in a CT scan.

## 3 RESULTS

### 3.1 Magnetite localization

**Figure 7:**
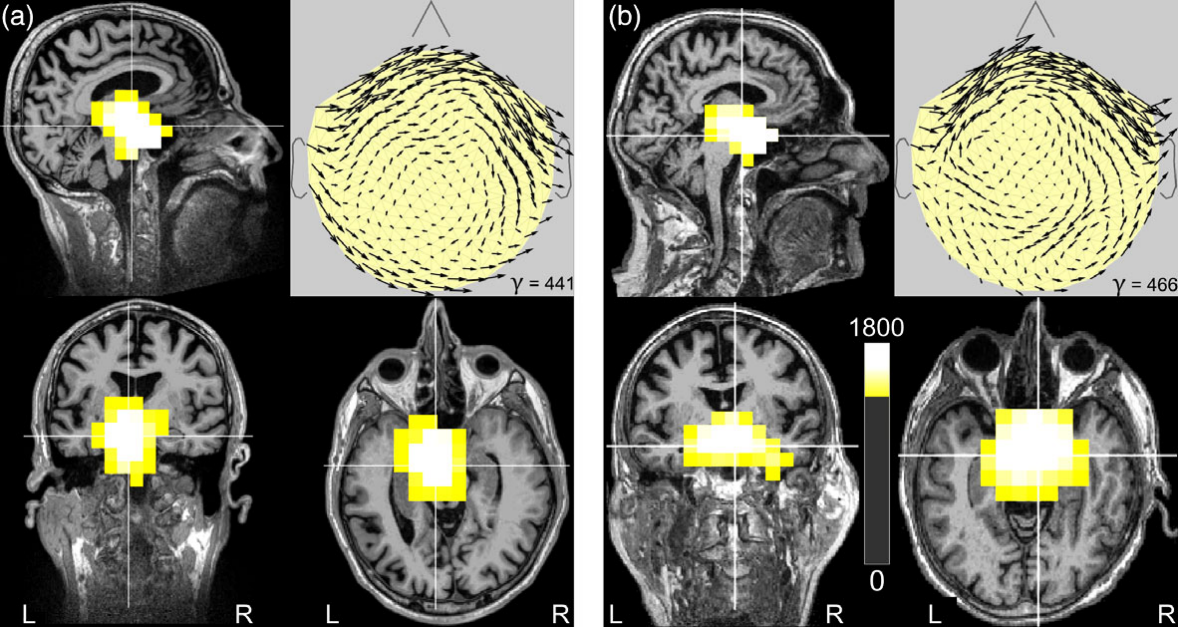
Arrow maps and magnetite locations of the two oldest subjects. Not shown here are the magnetite masses, calculated separately, but which we placed in Figure 8. (a) Upper right: Arrowmap of the particles of the 81 y/o subject. Other three images of a: Inverse solution placed onto the MRI of that subject. (b) The same for the 89 y/o subject. The location of magnetite particles is seen to be similar for these older disease‐free men, as well as their total mass, seen in Figure 8 to be about 12 μg [Color figure can be viewed at http://wileyonlinelibrary.com]

Localization in two of the subjects is shown in Figure 7 (step 4), along with the arrowmaps. It appears that, in these two subjects, the magnetite is single‐clumped; the clump is localized mostly to the limbic system regions, extending from the septum and forebrain, caudalward through the hypothalamus to the ventral midbrain. It is likely that the hippocampus is also a source of the signal. However, it is not known if the spread of the source is actual magnetite, or simply error of localization (see error discussion below). In the other nine cases, as the subjects get younger, the magnetite splits into two or three clumps, as is allowed by the distributed inverse solution. The color‐bar in Figure 7 represents an F‐score (Dale et al., 2000); this is a statistical distance for magnitude of magnetic dipoles with respect to the null distribution computed from empty room MEG recording. In order to be comparable across all subjects, we have used single 10‐min empty‐room recording for all subjects. Similar displays of three other subjects are shown in Supporting Information Figures S1–S3**.**

### 3.2 Magnetite mass versus age

**Figure 8:**
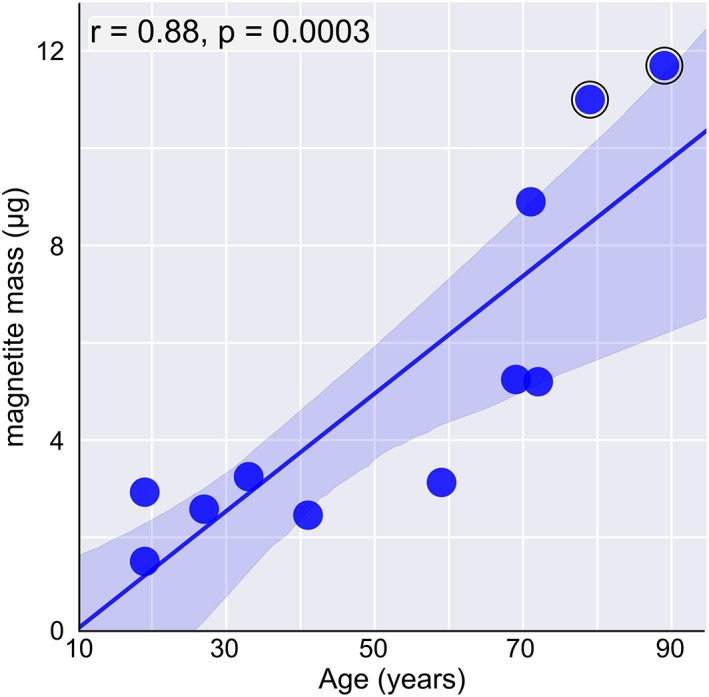
Result of our 11‐man study, showing total mass of magnetite vs. age of theses healthy male subjects. The two oldest subjects, in Figure 7, are represented by a larger circle. The mass is seen to increase with age, especially above 60 years. The maximum mass of magnetite is seen to be 11.8 μg, for the 89 y/o subject. The location of that mass is seen in Figure 7 to be in the area around the hippocampal formation [Color figure can be viewed at http://wileyonlinelibrary.com]

Figure 8 shows the final data of our study. There is increasing mass of magnetite with age. The scatter of the data points should be considered as partly experimental error (see Discussion), or actual variation of mass. As we refine our method, we expect in future that the error will be reduced, resulting in less scatter. Results here depend heavily on the calibration curve, where errors do need to be reduced. In any case, the increase of mass with age is beyond the present experimental error, shown by the r and p values, certainly for ages greater than 60.

We did one exploratory trial using a 1.2 T open‐MRI with magnetization in the x‐direction, on the 89 y/o subject, yielding approximately the same amount and distribution of magnetite, as with *z*‐magnetization, as expected.

### 3.3 Relaxation

We present some experimental results. As mentioned, the arrow maps such as those in Figure 7, were not only measured at 3 min. After magnetization but also about every 10–20 min after that, to look for changing and decreasing arrow patterns. Further, in four or five cases, arrow maps were also made 1 or 2 days later, and even later. It was found that there was usually some relaxation in the time interval 3–15 min after magnetization, perhaps about 10%, then slower change during the first day. There was only slow relaxation in the days ahead. However, this varied widely between subjects, and the general decrease was accompanied with complex changes in arrow patterns. After a month, in the 89 y/o subject, the arrows were reduced to about ½ in length. We never saw rapid relaxation.

We also attempted to see what happens during the first 3 min, with the oldest subject. That is, we performed a “rapid” measurement, looking at relaxation before 3 min. We used a heavy hand‐held permanent magnet for magnetization instead of the MRI magnet; it had a strength of about 0.03 T at a location 7 cm into the head, at perhaps 20% of saturation. This magnetization took place just outside the shielded room, allowing the first rapid dcMEG measurement at about 5 s, then every 20 s or so, thereafter. Although significant magnetization was seen, there was no relaxation, indicating at least there would have been no dramatic relaxation in the first few minutes, that we have missed with our standard MRI method. However, with greater magnetizing strength, there could have been some minor relaxation that we missed, say 3 or 5%. Perhaps we here activated a different range of particles.

## 4 DISCUSSION

### 4.1 Errors

We discuss the errors in these results, essentially the scatter in mass, in Figure 8. We aim to reduce these errors, but our goal at present is simply that the method be good enough to see gross phenomena. We plan the refinements for later. One source of error is our algebraic choice of γ, the square‐root system. We can estimate the error by calculating γ forward and varying the two angles about the *z*‐axis; ideally the measure would be defined such that it is independent of angle. Our calculations to date indicate an rms error of about 5%, for the physical quantity of mass, due to our definition of γ.

Another source of error is the relaxation after magnetization, say about 3%, as an estimation that we mentioned earlier. We used no correction as yet, for this phenomenon. Yet a third source of error were healthy hair follicles in some of the younger subjects, when touched against the fiberglass dewar wall during measurement. We here guess, again, an rms error of 5% for subjects younger than 50 y/o. All in all, we estimate an rms error of about 10% to be responsible for some of scatter of the dots. This should be factored into the data of future studies, for subjects with healthy hair.

The concept of spatial resolution perhaps should be discussed at this point. Considered again: how accurately are the particles located? At this early stage of method development, we accept a modestly poor resolution, with the understanding that out technique will improve, and the errors will be reduced, perhaps to an ultimate resolution of perhaps ±5 mm (similar to the MEG, containing 3 mm error due to co‐registration).

### 4.2 Relaxation and particle sizes

Concerning the size of the magnetite particles, our method is not good presently, for determining this quantity. Magnetite particle sizes less than 30 nm in diameter are superparamagnetic, therefore relax in times less than 1 s after magnetization, hence nanoparticles are not seen by us. Particles greater than 200 nm are multi‐domain, hence relax to a large degree as domain walls move, in somewhat longer times, with eventual much reduced magnetization. We should see these particles with reduced magnetization, and a little of their relaxation. Therefore, we probably see some multi‐domain particles. When we see some relaxation in the hours–day range, we do not know if that is true relaxation of multi‐domain particles, or some elasticity or other effect of brain tissue. All in all, it appears that we see mostly single domain particles in the 30–200 nm range. Our data does not strongly prove this, where we would need numerous measurements at different magnetizing strengths, that is, a variety of magnetization curves.

### 4.3 A variable magnet

In that regard, we note the possibility of gathering extra information by varying the magnetizing field. For example, the direction of the magnetizing field need not be the *z*‐direction, but with special magnets, can be the x and y directions as well. The *z*‐direction was chosen here because of the convenient proximity of our 1.5 T MRI. As noted in Results, our trial with magnetization in the x‐direction, yielded approximately the same amount and distribution of magnetite, as with *z*‐magnetization. Variation of the strength of the magnetizing field, should yield information on magnetite sizes. What is needed is a good, variable magnet. With this, a wide range of technology awaits. Eventually the physics says that the state of a particle group magnetized should be labeled by strength, mass, elasticity, and susceptibility. Much potential information concerning magnetite in the brain, is thus allowed with a good variable magnet, and its associated technology.

### 4.4 Particle locations

Considering the low resolution of our present method, in the older subjects we have seen that the magnetite could be in the upper brainstem (diencephalon and midbrain), the rostrally adjacent basal forebrain, or in the hippocampal formation; perhaps in any or all of those regions. This is in rough agreement with the findings in Gilder et al. (2018), where the brainstem and surrounding regions contained the most magnetite. In our younger subjects, with less total mass, the locations are less certain.

### 4.5 Source of magnetite

We now consider the origin of magnetite in the human brain. Particle sizes have been determined, in post‐mortem experiments, from the smallest superparamagnetic size to that of the single domain (Kirschvink, Kobayashi‐Kirschvink, & Woodford, 1992a; Schultheiss‐Grassi, Wessiken, & Dobson, 1999). Most authors propose that magnetite in the brain is a result of internal biological activity, some say with a definite biological purpose (Kirschvink, Kobayashi‐Kirschvink, & Woodford, 1992a; Kobayashi & Kirschvink, 1995). On the other hand, there is a claim (Maher et al., 2016) that magnetite nanoparticles come from external sources via the olfactory system, implying that Alzheimer's disease can perhaps have an external dust involvement. There is also a report that particles of another element (manganese) enter the brain in a similar way (Thompson et al., 2007), lending support to this magnetite route. In our study of healthy men, the data does not give support to either of these two views, because we do not see nanoparticles and are generally unsure of our sizes. Perhaps later, via our method, the question of origin can be answered, by finding particle locations in the living brain, with more accuracy, and their sizes.

### 4.6 Mass versus age

We next discuss the increase of magnetite mass with age and compare this with other reports. One early extensive report (Hallgren & Sourander, 1958) showed nonhaemin iron in different parts of the post‐mortem brain, usually increasing with age everywhere, but without separation of magnetite; however, it is not clear that magnetite would behave similarly. In another report (Harder et al., 2008) MRI was used to look at deep iron compounds as a function of age; these again increased with age, but again magnetite was not separated out. In the recent measurement of magnetite distribution in the post‐mortem brain, (Gilder et al., 2018) see no effect of age, and their total mass is less than ours by an order of magnitude. Perhaps the sampling of particle sizes is different. However, a prolific investigator of these phenomena is Prof. Jon Dobson and collaborators (Dobson, 2001, 2002, 2004) and they deal widely with magnetite mass and age, especially in Dobson (2004). In Figure 1a of this reference, their post‐mortem measurements show an increase of magnetite mass with age in older normal male subjects, with a total mass similar to what we measured in the live head. We thus agree with their results, which lends confidence to our method. Concerning the future, all in all, we might expect that older male patients with Alzheimer disease, would show greater than 20 μg, up a factor of two or more, from equivalent older normal subjects.

## 5 CONCLUDING REMARKS

The use of SQUIDs to look for magnetite in the brain is not new (Hautot, Pankhurst, & Dobson, 2005; Jia et al., 2008). However, these previous efforts were all post‐mortem. We offer the first in vivo method for using SQUIDs for the living brain, via the dcMEG. We ask: To what extent do we supply a tool for future understanding and diagnosis of neurodegenerative diseases? Our method for seeing magnetite is unique, and the ultimate diagnosis success will partly depend on the actual total mass increase of these particles and their locations, in Alzheimers' and other neurodegenerative diseases. This is to be determined in actual future studies of patients. We now understand how to deal with the artifact of hair follicles, and we are ready to study full‐haired male subjects, and female subject, and of course, patients. Thus, using this method allows various new studies of magnetite in the normal brain, and in the brain with neurodegenerative diseases as well.

## Supporting information


**Appendix S1:** SUPPLEMENTARY INFORMATION
