# Supplementary material for: Using the magnetoencephalogram to noninvasively measure magnetite in the living human brain
Source: Hum Brain Mapp. 2018 Nov 20;40(5):1654–65. doi: 10.1002/hbm.24477 (PMC6587731; doi:10.1002/hbm.24477)
Supplement: Supplementary file 1 — Appendix S1: SUPPLEMENTARY INFORMATION [file HBM-40-1654-s001.docx]

**SUPPLEMENTARY INFORMATION**

**Using the magnetoencephalogram to non-invasively measure magnetite in the living human brain**

Running title: Measuring magnetite in the living brain

Sheraz Khan^1, 2^ and David Cohen^1,2,3 *^

^1^Radiology, Massachusetts General Hospital, Harvard Medical School, Boston, MA, USA

^2^Athinoula A. Martinos Center for Biomedical Imaging, Boston, MA, USA

^3^Francis Bitter Magnet Lab, Massachusetts Institute of Technology, Cambridge, MA, USA

***Corresponding author:**

David Cohen, Ph.D.

Athinoula A. Martinos Center for Biomedical Imaging

Massachusetts General Hospital

149 13th Street

Boston, MA-02129, USA

Phone: +1 617-547-2658

Fax: +1 617-948-5966

E-mail: davcohen@mit.edu

1. **The expense of this new method**

In introducing our method, we note a negative factor: the difficult availability of the equipment, namely the dcMEG (and its shielded room), and the strongly magnetizing magnet. At present there are about 90 Elekta MEGs around the world, “ready to go” except for software additions, to convert MEGs to dcMEGs, as we did. Further, there are about 110 other MEGs around the world which could probably be altered by further software (using magnetometers instead of planar gradiometers if necessary) to be useful for these purposes. Therefore, for the easiest way to presently use our method, non-MEG investigators without an MEG must form alliances with any of the 90 Elekta-MEG groups. Otherwise, the cost of a completely new dc system is, unfortunately, about three million dollars; perhaps much less eventually, using new atomic or other non-SQUID magnetometers. On the positive side, there are about 200 MEG facilities presently available to enter this work. Not a small number, for a new technology. Recall the early days of PET.

Concerning the second item of equipment, the magnetizing field. If there is an MRI nearby, there is no problem, where the field is in the range 1-3 T. Otherwise a specialized dc magnet for the head must be obtained, at a cost of $20,000 to $50,000. However, this is a correct way to do it; to have an electromagnet where the field can be varied, to get hysteresis information, therefore much more particle information, as discussed previously.

1. **More subjects out of 11**


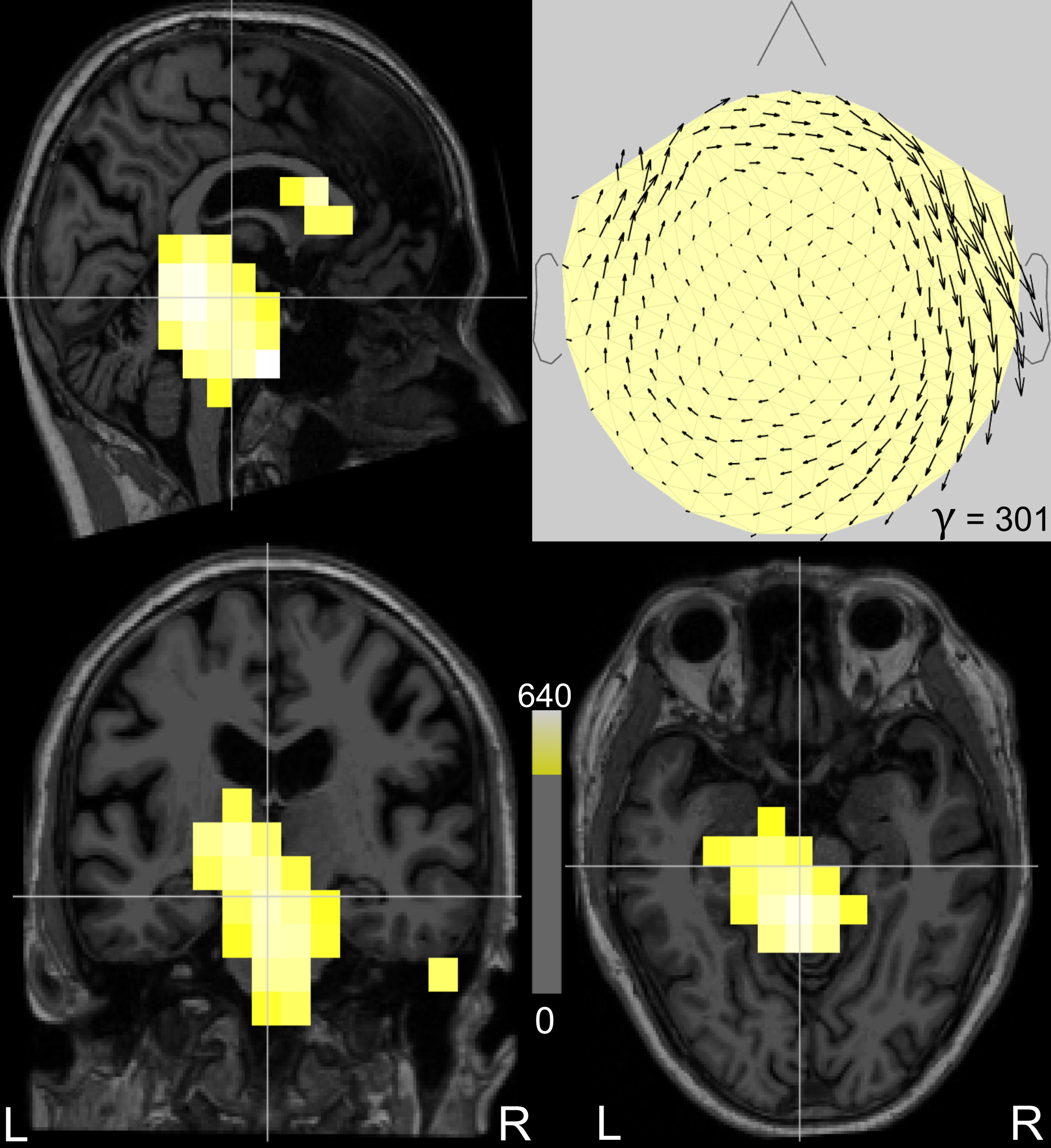


**Fig. S1.** Source image of 72 y/o subject. In his case there appear to be some spread of magnetite compared with the oldest two subjects.


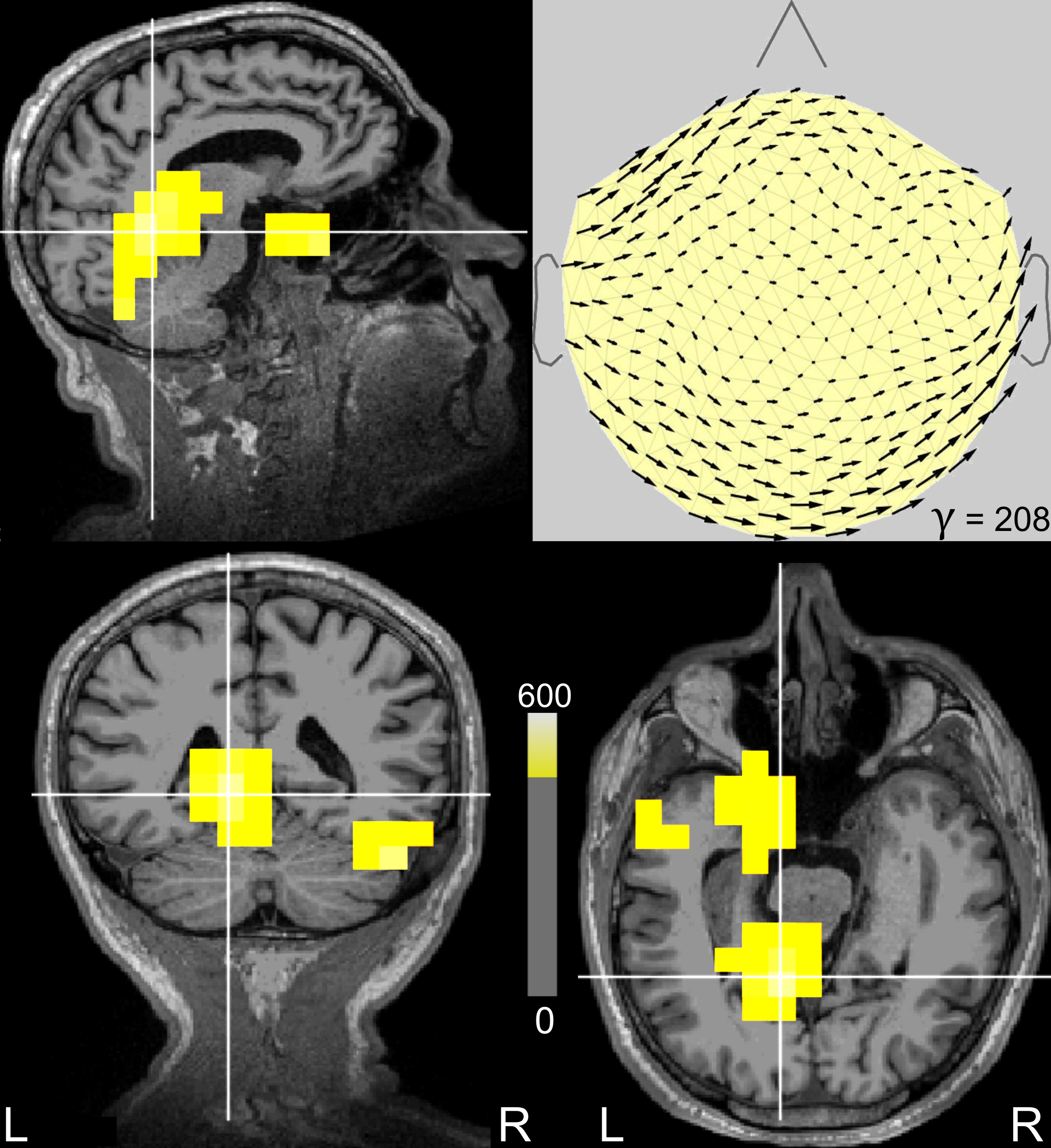


**Fig. S2.** Source image of 69 y/o subject. Again, there is some spread.


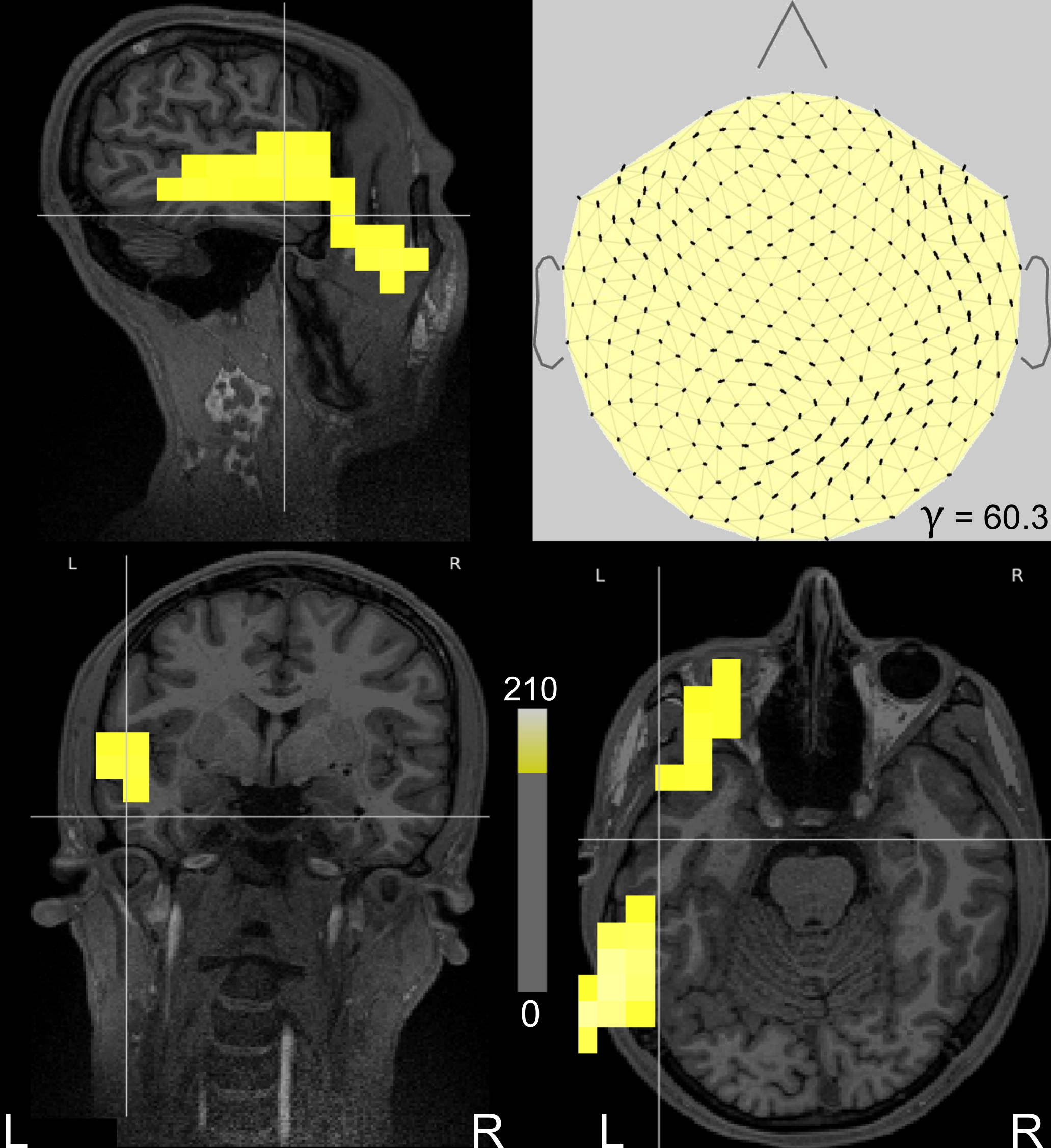


**Fig.S3.** Source image of 19 y/o subject. $\gamma$ is small for this youngest subject, indicating only a small mass of magnetite. His mass is the least of all. We don’t yet know if we are seeing mostly noise when the age is this young.
